# β-Lytic Protease of *Lysobacter capsici* VKM B-2533^T^

**DOI:** 10.3390/antibiotics9110744

**Published:** 2020-10-28

**Authors:** Alexey S. Afoshin, Mihail A. Konstantinov, Ilya Yu. Toropygin, Irina V. Kudryakova, Natalia V. Vasilyeva

**Affiliations:** 1 Laboratory of Microbial Cell Surface Biochemistry, G.K. Skryabin Institute of Biochemistry and Physiology of Microorganisms, Russian Academy of Sciences, FRC PSCBR RAS, 5 Prosp. Nauki, Pushchino, Moscow Region 142290, Russia; alex080686@mail.ru (A.S.A.); kudryakovairina@yandex.ru (I.V.K.); 2 Department of Proteomics, V.N. Orekhovich Research Institute of Biomedical Chemistry, Russian Academy of Medical Sciences, 10 Pogodinskaja Str., Moscow 119832, Russia; mishanyamihail@ya.ru (M.A.K.); toropygin@rambler.ru (I.Y.T.)

**Keywords:** β-lytic protease, specificity, antimicrobial activity, *Lysobacter*

## Abstract

Bacteriolytic enzymes are promising antimicrobial agents for developing new-generation drugs. Recently, we have isolated a β-lytic protease (BlpLc) from the culture liquid of *Lysobacter capsici* VKM B-2533^T^. This BlpLc possesses a valuable property, not described for β-lytic proteases (Blps) earlier, of hydrolyzing living cells of *Staphylococcus aureus* 55 MRSA clinical isolate. This work phylogenetically characterized the BlpLc and investigated its properties. Analysis revealed a variability of pre-/pro-parts of Blp precursors. The mature BlpLc is the closest to the earlier annotated but not isolated Blp from *Lysobacter* sp. Root690. The biochemical characterization found conditions for the BlpLc general bacteriolytic activity relative to autoclaved *S. aureus* 209P cells to differ from that of earlier isolated Blp. Unexpected was the effect of serine (phenylmethylsulfonyl fluoride (PMSF)) and cysteine (p-chloromercuribenzoate (p-CMB)) protease inhibitors on BlpLc bacteriolytic and proteolytic activities. The specificity of BlpLc proteolytic action relative to hemoglobin, elastin, gelatin, collagen, azofibrin, myoglobin, ovalbumin, and ovamucoid was found. New types of peptide bonds—Gly-X, Ser-X, Lys-X, Ala-X, Val-X, Glu-X, and Phe-X—hydrolyzed by the enzyme in protein substrates were first revealed using MALDI-TOF. Turbidimetrically, the BlpLc was found to lyze living cells of *S. aureus* 209P, *Micrococcus luteus* B1819, and *M. roseus* B1236, which is important for expanding the enzyme’s applied properties.

## 1. Introduction

In 2022, it will have been a century since the publication of the first article [1] about the first bacteriolytic enzyme, lysozyme. Since the time of its discovery and for over about 50 years, bacteriolytic enzymes had not been the subject of scientific interest. It was only starting from the 1970s that publication activity in this respect began to rise. The issue of pathogen antibiotic resistance started to be recognized and, in this context, the need emerged to search for new antimicrobial agents.

At present, lysozyme is one of the most investigated proteins. This enzyme is the basis of many antimicrobial drugs. However, its action is limited to a minor range of bacteria, mainly *S. aureus*, *M. luteus*, *Bacillus stearothermophilus*, and *Clostridium tyrobutyricum* [2,3,4]. To date, modified forms of lysozyme have been developed, with a broader spectrum of lytic action, including Gram-negative bacteria [4]. In bacterial cell walls, lysozyme degrades the bond between N-acetyl glucosamine and N-acetylmuramic acid residues of peptidoglycan, exhibiting the so-called muramidase activity. Besides muramidases, there are also other bacteriolytic enzymes as follows: amidases that hydrolyze the bond between the first amino acid of the peptide subunit and N-acetylmuramic acid; endopeptidases breaking the peptide bonds in the peptide stem and the interpeptide bridge of peptidoglycan’s peptide subunits; and glucosaminidases that digest the same bond as muramidases but to release N-acetyl glucosamine at the reducing terminus [5]. 

All living organisms produce bacteriolytic enzymes. In bacteriophages, these enzymes are called endolysins. In bacteria, bacteriolytic intracellular enzymes (autolysins) are implicated in cell growth and division. Extracellular bacteriolytic enzymes provide the cell with nutritive matter and ensure the assimilation and expansion of ecological niches. All bacteriolytic enzymes of bacteria are promising for use in medicine as the base of novel antimicrobial drugs. 

Among bacteria, representatives of the genus *Lysobacter* are known to possess high lytic activities. This is due to their production of complexes of biologically active compounds attributed to various classes, for example, extracellular enzymes (proteases, bacteriolytic enzymes, glucanases, chitinases), short peptides (cyclo(L-Pro-L-Tyr)), and antibiotics [6,7,8,9,10,11]. 

Research into antimicrobial compounds produced by *Lysobacter* has a history. The antibiotic myxin [12] and bacteriolytic enzymes α-lytic protease and Blp [13] were the first isolated biologically active agents in *Lysobacter*. These are the second and third revealed and isolated bacteriolytic enzymes after lysozyme. They are the first bacteriolytic enzymes found in bacteria. Producers of these compounds were attributed at the time to unidentified species of myxobacteria *Sorangium* sp. When the genus *Lysobacter* was formed in 1978, it included the producer of these compounds [14], now known as *L. enzymogenes.* To date, the amidase CwhA of *L. enzymogenes* [15] and five lytic proteins of *Lysobacter* sp. XL1, among which the proteases L1, L4, and L5, amidase L2, and muramidase L3 [16,17,18,19,20], have been also isolated and partially characterized. Structurally, only the α-lytic protease of *L. enzymogenes* [21] and lytic proteases L1 and L5 of *Lysobacter* sp. XL1 [22,23] have been characterized.

More than fifty years have passed since the time when the first agents were isolated; the most investigated bacteriolytic enzyme of *Lysobacter* is the α-lytic protease. The Blp isolated at the same time has been less studied. It is known that this protein is a Zn-dependent metalloproteinase. Besides substrates for proteases, it hydrolyzes a number of Gram-positive and Gram-negative microorganisms [24,25]. The biochemical properties of Blps isolated in different *Lysobacter* species have been described not completely, interaction of the protein with target cells has not been studied, the spatial structure has not been resolved, and the prospects of using the protein in medicine have not been investigated. 

Studies of the *L. capsici* VKM B-2533^T^ lytic potential enabled the isolation of bacteriolytic enzymes, among which the BlpLc (GenBank accession number QGQ32939.1) was identified [26]. The enzyme possesses a proteolytic activity with respect to casein and a bacteriolytic activity with respect to autoclaved staphylococcal cells. Besides that, the protein has a potent lytic action with respect to methicillin-resistant living staphylococcal cells. A similar action with respect to *Staphylococcus* has not been described earlier. Thus, the aim of this work was to characterize the BlpLc. 

## 2. Results

### 2.1. Phylogenetic Analysis 

Sequencing of the cloned BlpLc sequence conducted earlier [26] enabled the determination of the amino acid composition and functional elements of the protein. The BlpLc is synthesized as a large precursor, a prepro-protein. Its total length is 378 aa; the length of the pre-part (signal peptide) is 26 aa, the pro-part is 173 aa, and the mature part of the protein is 179 aa. 

**Figure 1:**
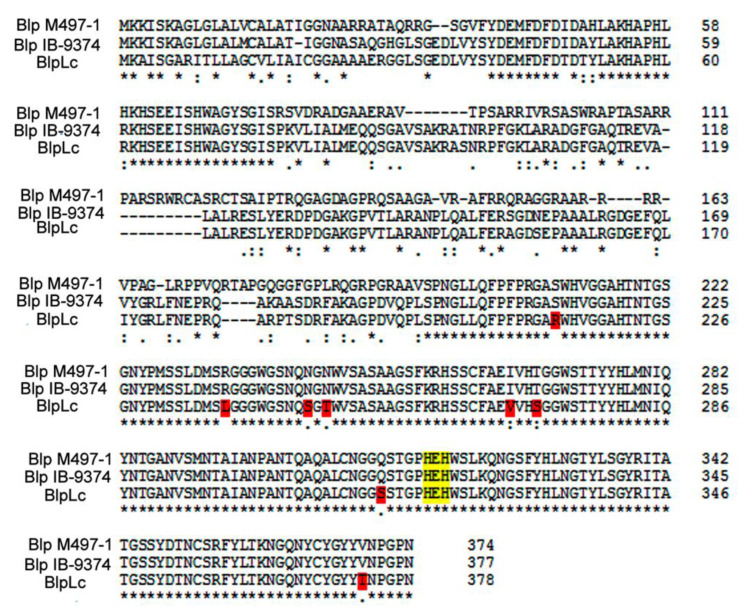
Multiple alignment of amino acid sequences in the full length of the Blps. The BlpLc pre-part, 1–26 aa; the pro-part, 27–199 aa; the mature part, 200–378 aa. Yellow highlights, HxH motifs characteristic of M23 family proteases. Red highlights, differences between amino acid compositions of the BlpLc mature part and of the earlier isolated and characterized Blps from *L. enzymogenes* M497-1 and *Lysobacter* sp. IB-9374.

Multiple alignment of the BlpLc precursor with the precursors of the earlier isolated and characterized Blps from *L. enzymogenes* M497-1 and *Lysobacter* sp. IB-9374 showed that the mature part of the proteins had a high identity of 96%. Herewith, the pre- and pro-parts can differ significantly (Figure 1). 

The structure of the Blp signal peptide is represented by the following three regions: the positively charged N-region MKAISGARI responsible for the interaction with the negatively charged membrane; the hydrophobic H-region TLLAGCVLIAIC implicated in membrane association and translocation; and the C-region GGAAA containing the signal peptidase recognition site (Table 1).

Analysis showed the BlpLc to have a lower charge of the N-region but a high hydropathy (hydrophobicity) of the H-region as compared with signal peptides from *L. enzymogenes* M497-1 (earlier identified as *Achromobacter lyticus*) [27] and *Lysobacter* sp. IB-9374.

The BlpLc pro-part is 95% identical to the Blp pro-part of strain IB-9374 and significantly differs from the Blp pro-part of strain M497-1 (the query coverage, a mere 23%). The pro-part of M497-1 Blp is enriched with arginine, which has been earlier noted in [25]. Herewith, the pro-parts of strain IB-9374 Blp and of the BlpLc are devoid of similar arginine repeats. 

The mature part of the BlpLc includes a conserved motif HxH characteristic of M23 family proteases. Attention in this mature part is drawn to such amino acid substitutions as arginine for serine and leucine for arginine. These substitutions can be of significance for the manifestation of the BlpLc physico-chemical properties. 

**Figure 2:**
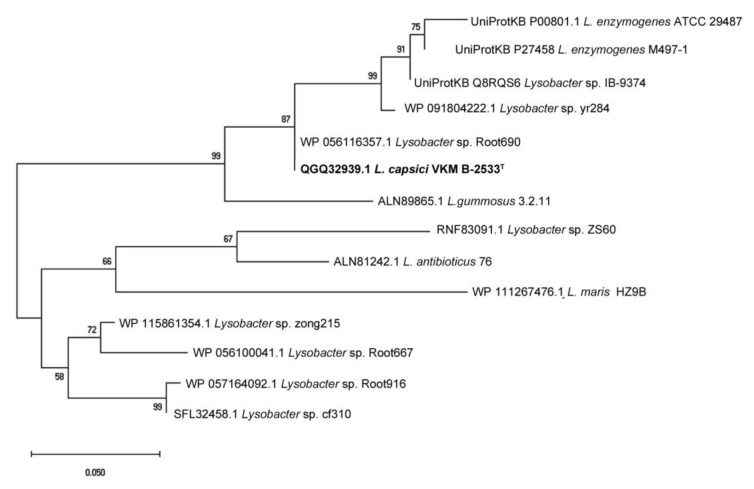
Dendrogram of Blps of *Lysobacter* bacteria.

The dendrogram in Figure 2 shows both the earlier characterized enzymes and annotated closest analogues from the genus *Lysobacter* (the value of *E* < 2 × 10^−93^). It can be seen that the proteases are clustered into two groups. One group is represented by the protease from *L. antibioticus* 76 and other less investigated species. The other group is represented by the protease from *L. enzymogenes* M497-1. The protease annotated in *Lysobacter* sp. Root690 clusterizes the most closely to the protease from *L. capsici* VKM B-2533^T^. Thus, it follows that at the moment there are no isolated and characterized, most phylogenetically close, homologues for the BlpLc.

### 2.2. Physico-Chemical Properties of BlpLc 

Optimal conditions for revealing the bacteriolytic activity of the BlpLc were investigated with respect to autoclaved staphylococcal cells. It was found that the optimal conditions were pH 9.0; buffer concentration, 5.0 mM; temperature, 50 °C. Half-inactivation temperature of the enzyme was 57 °C. The enzyme was stable at pH from 4 up to 7 and from 10 to 11 (Supplementary Materials: Figures S1 and S2). 

Experiments were carried out to determine the impact of NaCl, metal ions, and metal substitution in the active site of the enzyme. 

**Figure 3:**
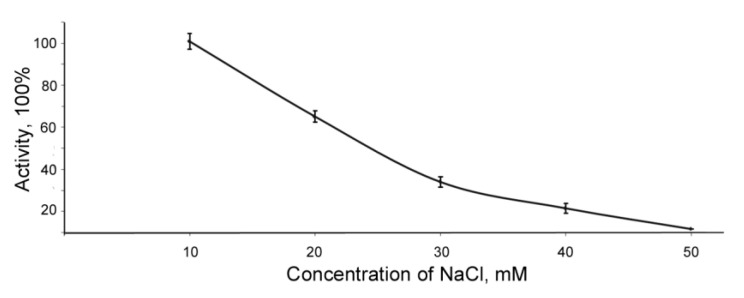
Effect of NaCl on the bacteriolytic activity of BlpLc. Values represent means ± standard deviations of three independent measurements.

The activity of the BlpLc was shown to decrease sharply at an increase of the concentration of NaCl (Figure 3). 

Metal ions inhibited the enzyme’s bacteriolytic activity to various degrees. Zn^2+^, Cu^2+^, and Fe^2+^ ions had the greatest effect (Table 2). Metal substitution experiments showed that Zn^2+^ ions restored the bacteriolytic activity by 75%. Addition of divalent ions of Ni, Cu, Mn, Mg, and Ca did not lead to enzyme activity recovery. 

It was found that in the presence of a 1% Triton X-100 solution the activity of the enzyme was preserved. At the same concentration of SDS and H_2_O_2_, the activity decreased by half. A further increase of the concentration of the agents led to a strong inhibition of enzyme activity (Table 3). 

On the whole, the physico-chemical properties of BlpLc are similar to those of the earlier isolated and characterized proteases. Still, some differences are there. The optimal ionic strength of enzymatic hydrolysis of autoclaved staphylococcal cells was found to be lower. The BlpLc was first shown to be capable of operating in the presence of detergents and bleacher. 

### 2.3. Effect of Inhibitors on BlpLc Activity

The results of the inhibitor analysis are given in Table 4. The analysis showed that the BlpLc was inhibited by 1.10-phenanthroline by 100%, which is characteristic of Zn-dependent metalloproteases. Unexpected proved to be the results using serine (PMSF) and cysteine (p-CMB) protease inhibitors. It was found that 5 mM p-CMB and 10 mM PMSF totally inhibited the BlpLc, which is not typical of metalloproteases. Initially, we suggested this to be due to substrate specificities (autoclaved staphylococcal cells were used). However, additional experiments showed that the enzyme stopped to hydrolyze casein after the treatment with PMSF and p-CMB (data not shown). Probably, serine and cysteine occur in the composition of the enzyme’s functional groups with which the inhibitor interacts. More experiments, including structural studies, are required to prove this. 

### 2.4. Determination of BlpLc Specificity of Action and Hydrolyzed Bond Type

**Figure 4:**
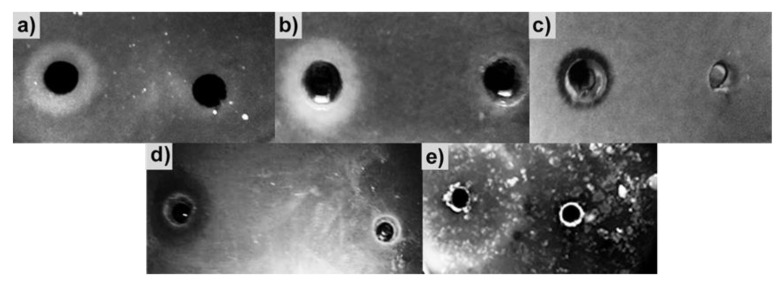
Specificity of BlpLc action with respect to different substrates: (**a**) gelatin, (**b**) azofibrin, (**c**) elastin, (**d**) hemoglobin, and (**e**) collagen.

Specificity of action of the enzyme was determined with respect to substrates characteristic of proteases. Using the spot test, the BlpLc was shown to hydrolyze hemoglobin, elastin, gelatin, collagen, and azofibrin (Figure 4). 

The type of hydrolyzed bond was determined by MALDI-TOF using myoglobin, hemoglobin, ovalbumin, and ovamucoid as substrates (Figure S2, Supplementary Materials). 

The BlpLc hydrolyzed the Gly-X bond in all substrates. In some substrates, the enzyme also digested the Ser-X, Lys-X, Ala-X, Val-X, Glu-X, and Phe-X bonds (Figure S2, Supplementary Materials). However, not all bonds of this type that occur in the substrate were hydrolyzed. 

### 2.5. Antimicrobial Action of BlpLc

Earlier, the enzyme was shown to lyze methicillin-resistant staphylococcal cells with high efficiency [26]. In this work, we investigated its action with respect to other Gram-positive bacteria, namely *S. aureus* 209P, *M. luteus* B1819, *M. roseus* B1236, and *B. subtilis* W23, and the Gram-negative bacterium *Escherichia coli* K12. The greatest bacteriolytic activity of the enzyme was found with respect to *M. luteus*, i.e., 69,500 ± 6364 LU/mL. The enzyme also lyzed another species of the *Micrococcus* genus, *M. roseus*, with an activity of 25.0 ± 4.2 LU/mL. This difference is, possibly, due to features of cell wall structure in these bacteria. The activity of the enzyme with respect to living cells of *S. aureus* 209P was 270.0 ± 2.8 LU/mL. With respect to *B. subtilis* W23 and *E. coli* K12, the enzyme was inactive. 

## 3. Discussion

The Blp is one of the first bacteriolytic enzymes found in bacteria. When an enzyme identified as the Blp was isolated from the culture liquid of *L. capsici* VKM B-2533^T^, it showed an earlier undescribed property, a potent staphylococcal action with respect to living methicillin-resistant staphylococcal cells. The results of a phylogenetic analysis carried out in this work showed that the BlpLc was the closest to an enzyme annotated but not isolated earlier in *Lysobacter* sp. Root690. Thus, the enzyme needed to be further characterized. 

Comparative characterization of the amino acid sequences of Blp precursors from *L. capsici* VKM B-2533^T^, *L. enzymogenes* M497-1, and *Lysobacter* sp. IB-9374 showed that the mature parts of the proteins were rather conserved. The pre- and pro-parts, however, were variable. This is of certain interest, because these parts are responsible for topogenesis of the proteins. According to the theory of protein topogenesis, the pre-part provides for secretion of the precursor via the cytoplasmic membrane into the periplasm [28]. Furthermore, the pro-part can be responsible for correct folding of the protein, as it has been shown for the α-lytic protease of *L. enzymogenes* [29]. To date, the secretory pathways in *Lysobacter* bacteria have not been investigated, and the revealed differences of Blp pre- and pro-parts are difficult to explain. Probably, these distinctions do not significantly affect the topogenetic processes. It can also be suggested that different species can have particular features of the interaction of proteins with the secretion apparatus. Further research is required to understand the revealed features.

Although the identity of Blp mature parts was 96%, the BlpLc was found to have amino acid substitutions that may affect the structural organization of the protein; this, in turn, may determine its catalytic features. 

Earlier isolated Blps have been characterized to various degrees (Table 5).

It can be noted that the common properties for the isolated Blps are alkaline conditions for the maximal bacteriolytic activity to be exhibited. The temperature optimum for the protease from *L. capsici* is lower, and the thermal stability is of the same order as in the earlier investigated enzymes. A characteristic distinction of this protease is the low value of ionic strength. This is also attested by the results of NaCl effect assays on enzyme activity. Experiments on the substitution of metal in the BlpLc active site showed that only Zn^2+^ ions restored the catalytic activity of the enzyme. This can indicate that Zn^2+^ is a required metal for the BlpLc catalytic activity to be exhibited. The results of pH stability tests showed the enzyme to be stable the least under conditions of optimal pH values. This can be explained by an increase of enzyme autolysis under the optimal conditions. This is a known phenomenon for proteases [31,32]. The observed distinctions in enzyme properties can be explained, on the one hand, by the different techniques of producing substrate used for analysis. To study the properties of bacteriolytic enzymes, our laboratory uses cells of autoclaved, washed, frozen, and then freeze-dried *S. aureus* 209P [19]. To study the properties of proteases from *Lysobacter* sp. IB-9374 and from *L. enzymogenes* M497-1, use was made of *S. aureus* or *M. luteus* cells prepared in a different way [24,25]. This may affect the availability of substrate for the enzyme and, correspondingly, its properties. On the other hand, this may also be due to existing structural differences. 

The possible significant structural distinctions can also be indicated to by the results of inhibitor analysis. The enzyme from *L. capsici* is inhibited by PMSF and p-CMB. This may indicate the occurrence of functional groups comprising Ser and Cys.

The specificity of action of the BlpLc was determined with respect to substrates for proteases, namely casein, elastin, gelatin, collagen, azofibrin, myoglobin, hemoglobin, ovalbumin, and ovamucoid. In some substrates, the types of hydrolyzed bonds were determined. This paper presents new sites of peptide bond hydrolysis besides the earlier established bonds hydrolyzed by other Blps (Table 6). Substrate hydrolysis was found to have some peculiarities, i.e., not all identical bonds in one substrate could be dehydrolyzed. As native proteins were used for this analysis as substrate, it can be assumed that bonds on the surface of the protein molecule were accessible for the BlpLc. The same bonds inside the protein molecule remained not dehydrolyzed. If the primary substrate specificities of staphylolytical proteases of the M23 family are compared, it can be seen that the Blps possess the broadest specificity spectrum (https://www.ebi.ac.uk/merops/).

The Blp was given its name for its ability to break down bacterial cells. For earlier isolated proteases, it has been shown that they break freeze-dried cells (Table 6). It should be noted that, based on the results of freeze-dried cells’ breakdown, it cannot be maintained that the enzyme will break living cells, too.

For the BlpLc, it was shown that the enzyme lyzed living cells of *S. aureus* 209P, *M. luteus* B1819, and *M. roseus* B1236 and failed to break cells of Gram-negative bacteria. Interestingly, the activity of the BlpLc was 2700 times as high with respect to *M. luteus* B1819 as with respect to *M. roseus* B1236. This can be due to distinctions in their cell wall structures. First, the peptidoglycans of these bacteria belong to different types [37]. Earlier, it was shown that in the peptidoglycan of *M. luteus* the Blp from *L. enzymogenes* hydrolyzes the bond D-Ala-L-Ala [24]. Given that, *M. roseus* has exactly the same bond at the same position, but the composition of the interpeptide bridge is generally different. An important difference is the occurrence of lipomannan in the cell wall of *M. luteus*, which in *M. roseus* is absent [38]. In this work, the sensitivity of *M. luteus* to lactoferrin was shown. Earlier, it was shown in our laboratory that teichoic acids can take part in the interaction of bacteriolytic enzymes with the *S. aureus* cell wall [39]. It is probable that, for the efficient hydrolysis of the peptidoglycan, bacteriolytic enzymes need a certain spatial structure of the cell envelope and/or the occurrence of certain components in it. Research into the interactions of bacteriolytic enzymes with the cell wall will in the future make it possible to find substances enhancing the operation of bacteriolytic enzymes as components of antimicrobial drugs. 

Thus, we characterized the antimicrobial agent of *L. capsici* VKM B-2533^T^ hydrolyzing micrococcal and staphylococcal cells. The antibacterial activity of the culture liquid of this bacterium is however much broader [26]. Moreover, the *L. capsici* VKM B-2533^T^ culture liquid possesses rather potent antifungal and yeast lytic activities. It is a matter of the future to establish lytic agents responsible for these activities. 

## 4. Materials and Methods 

### 4.1. Strain and Cultivation Conditions 

The culture of *L. capsici* VKM B-2533^T^ was grown on a KSP medium containing casein, 2.5 g/L; sucrose, 2.5 g/L; peptone, 2.5 g/L; KH_2_PO_4_, 0.1 g/L; K_2_HPO_4_, 0.1 g/L; MgSO_4_ 7H_2_O, 0.1 g/L; pH 7.2 [40] at 29 °C with aeration. Agarized media contained 1.5% agar. 

### 4.2. Isolation of BlpLc 

The BlpLc was isolated from the culture liquid of *L. capsici* as described earlier [26]. 

### 4.3. Protein Concentration Assay 

The concentration of protein in preparations was determined by the Bradford method [41]. The reaction was run according to the protocol for Coomassie (Thermo Fisher Scientific, Waltham, MA, USA). The concentration was determined by the calibration curve plotted for an aqueous solution of BSA (Sigma-Aldrich, St. Louis, MO, USA) within the range of 1–25 μg mL^–1^. 

### 4.4. Determination of BlpLc Bacteriolytic and Proteolytic Activity

The bacteriolytic activity of the BlpLc was determined turbidimetrically using autoclaved or living cells of *S. aureus* 209P as well as living cells of other Gram-positive bacteria, namely *M. luteus* B1819, *M. roseus* B1236, *B. subtilis* W23, and *E. coli* K12. An enzyme preparation in the amount of 100 μL (1.7 μg) was added to a 0.900-mL suspension of cells in 0.01 Tris-HCl, pH 8.0, with an optical density of 0.5 o.u. at 540 nm; the mixture was incubated at 37 °C for 10 min. The reaction was stopped by placing test tubes into ice; the absorption of the suspension at 540 nm was measured. An amount of enzyme, which led to a decrease of absorption of the cell suspension by 0.01 o.u. at 37 °C per 1 min, was taken to be a unit of bacteriolytic activity (LU). 

The lytic activity of the BlpLc (2.3 μg) with respect to *B. subtilis* W23 and *E. coli* K12 was also analyzed by counting CFU/mL during the seeding of cells into an agarized medium. 

The proteolytic activity was measured by the rate of casein splitting according to Hull’s method [42]. 

### 4.5. Electrophoresis of Proteins

The electrophoresis was conducted in 12% SDS-PAGE by the Laemmli method [43]. Proteins were visualized in gel using Coomassie Brilliant Blue R-250. SM0431 (Thermo Fisher Scientific, Waltham, MA, USA) was used as molecular weight markers. 

### 4.6. Determination of Conditions for Optimal Manifestation of BlpLc Bacteriolytic Activity 

The optimal conditions for revealing the bacteriolytic activity of the enzyme were determined with respect to autoclaved *S. aureus* 209P cells turbidimetrically as described above. 

The optimal values of pH, buffer concentration, temperature, half-inactivation temperature, and enzyme stability pH were determined by the standard techniques using a Britton–Robinson buffer. 

Assays of metal effects on the enzyme activity were carried out using a 5-mM Britton–Robinson buffer, pH 9.0, containing 1 mM CaCl_2_, ZnCl_2_, CuSO_4_, NiSO_4_, FeSO_4_, MgCl_2_, and MnCl_2_. The reaction mixtures were incubated at 50 °C, then the bacteriolytic activities were determined. 

The effect of NaCl on the enzyme activity was determined using a 5-mM Britton–Robinson buffer, pH 9.0, containing NaCl at concentrations of 10 to 50 mM. The mixtures were incubated at 50 °C, then the bacteriolytic activities were determined. 

### 4.7. Effect of Inhibitors on BlpLc Activity 

To study the effect of inhibitors on the enzyme activity, we used metalloprotease inhibitors EDTA and 1,10-phenanthrolin at concentrations of 10 and 50 mM and of 1, 2.5, and 5 mM, respectively; serine protease inhibitor PMSF, 5 and 10 mM; cysteine protease inhibitor p-CMB, 1 and 5 mM. Analysis was carried out using a 5-mM Britton–Robinson buffer, pH 9.0. An inhibitor was added to 4 μL (41.89 μg/mL) of the enzyme, and the volume was adjusted by a 5-mM Britton–Robinson buffer to 0.3 mL. The mixture was incubated at room temperature for 15 min, 0.3 mL substrate was added, and the residual bacteriolytic activity and proteolytic activity at 37 °C were determined. 

### 4.8. Determination of the Effect of Metal Substitution in the Enzyme Active Site

The effects of metal substitutions in the active site of the enzyme on the activity were determined spectrophotometrically using autoclaved *S. aureus* 209P cells as substrate. The substrate was dissolved in a 5-mM Britton–Robinson buffer, pH 9.0, to an optical density of OD_540_ = 0.9. 1,10-Phenanthrolin to a final concentration of 5 mM was added to 2 μL (47.55 μg/mL) of the active enzyme (1464 LU/mL) to eliminate metal from the BlpLc active site; the volume was adjusted to 0.3 mL by a 5-mM Britton–Robinson buffer, pH 9.0. The mixtures were incubated at room temperature for 20 min. At this stage, the formation of the apoenzyme was monitored. Then, CaCl_2_, ZnCl_2_, CuSO_4_, NiSO_4_, MgCl_2_, and MnCl_2_ were added to a final concentration of 1 mM and the mixtures were incubated at room temperature for 10 min more. After this, 0.3 mL substrate each was added to the reaction mixtures, and the bacteriolytic activities were determined at 37 °C for 5 min. 

### 4.9. Assay of the Action of Bleacher and Detergents on BlpLc Bacteriolytic Activity

To study the effect of bleacher and detergents on the activity of the enzymes, use was made of the following: ionogenic detergent SDS, at concentrations of 1 and 5 mM; nonionogenic detergent Triton X-100, at 1 and 5 mM; and hydrogen peroxide H_2_O_2_ as a bleacher, at 1 and 5 mM. A detergent or bleacher and a 5-mM Britton–Robinson buffer, pH 9.0, were added to 4 μL (41.89 μg/mL) of the enzyme; the volume was adjusted to 0.3 mL. The mixture was incubated at room temperature for 15 min, 0.3 mL substrate was added, and the residual bacteriolytic activity was determined at 37 °C for 10 min. 

### 4.10. Determination of BlpLc Substrate Specificity by Spot Test

The substrate specificity was determined with respect to gelatin, azofibrin, collagen, elastin, and hemoglobin. Amounts of a 50-μL preparation and a buffer as a control were introduced into wells made in Petri dishes containing a 0.5–1% substrate in a 5-mM Britton–Robinson buffer, pH 9.0, and 1.5% agar. The dishes were incubated at 29 °C up to the appearance of clarification zones. 

### 4.11. Determination of the Type of Hydrolyzed Bond in Protein Substrates by MALDI-TOF 

The site specificity of the BlpLc was determined using commercially available native proteins, namely myoglobin, hemoglobin, ovalbumin, and ovamucoid. The hydrolysis products were identified by MALDI-TOF and tandem MALDI-TOF/TOF using a Bruker Ultraflex II mass spectrometer (Bruker, Bremen, Germany). 

Hydrolysis was conducted in a 100-mM ammonium bicarbonate buffer, pH 7.8, or in a 5-mM Britton–Robinson buffer, pH from 8.8 to 9.2. The reaction mixture contained 56 μg/mL enzyme and 400 μg/mL substrate. The mixture was incubated for 5, 15, 30, 60, and 120 min at 37 °C. The spectra were recorded using 2.5-dihydroxybenzoic acid (DHB). 

### 4.12. Phylogenetic Analysis 

Multiple alignment of the amino acid sequences of the Blps from *L. enzymogenes* M497-1, *Lysobacter* sp. IB-9374, and *L. capsici* VKM B-2533^T^ was conducted in Clustal Omega 1.2.4 (https://www.ebi.ac.uk/Tools/msa/clustalo/) [44]. The functional analysis of the proteases was carried out using the MEROPS server 12.3 (https://www.ebi.ac.uk/merops/) [45]. The dendrogram of the proteases was plotted in MEGA 10.0.5 [46]. Analysis was done using the statistical method of maximum likelihood estimation and the Jones–Taylor–Thornton amino acid substitution model [47]. The bootstrap test was conducted with the number of iterations of 1000 [48]. The search for nearest identical proteins was carried out using Basic Local Alignment Search Tool (https://blast.ncbi.nlm.nih.gov/Blast.cgi) [49]. Analysis of signal peptide structures was conducted using the Phobius server (http://phobius.sbc.su.se/) [50]. Hydropathy was calculated using a Kyte and Doolittle scale using the bioinformation server at http://www.bioinformatics.org/sms2/protein_gravy.html [51]. 

## Figures and Tables

**Table 1:** Organizations of Blp signal peptides compared *.

Protease	Region					Signal Peptide Length
	N		H		C	
	Length, aa	Net Charge	Length, aa	Hydropathy Index	Length, aa	
*L. capsici* VKM B-2533^T^ GenBank no. QGQ32939.1	9 MKAIS GARI	+2	12 TLLAGCVLIAIC	2.675	5 GGAAA	26
*Lysobacter* sp. IB-9374 UniProtKB Q8RQS6	6 MKKISK	+3	12 AGLGLALMCALA	2.167	8 TIGGNASA	26
*L. enzymogenes* M497-1 UniProtKB-P27458	6 MKKISK	+3	12 AGLGLALVCALA	2.358	12 TIGGNA ARRATA	30

* Comparison is only made against the earlier isolated proteases, for which the amino acid sequences of signal peptides are available.

**Table 2:** Effect of metals on BlpLc activity.

Metal salt	Inhibition, %
CaCl_2_	25.0 ± 2.5
ZnCl_2_	93.0 ± 1.4
CuSO_4_	99.10 ± 0.07
NiSO_4_	54.5 ± 5.4
FeSO_4_	69.4 ± 3.0
MgCl_2_	28.7 ± 3.2
MnCl_2_	39.2 ± 2.7

**Table 3:** Effect of bleacher and detergents on BlpLc activity.

Agent	Concentration, %	Residual Activity, %
SDS	1	54.9 ± 3.7
	5	0
Triton X-100	1	109.0 ± 2.3
	5	10.9 ± 1.9
H_2_O_2_	1	67.0 ± 1.3
	5	26.0 ± 2.1

**Table 4:** Effect of inhibitors on BlpLc activity with respect to autoclaved cells of *S. aureus* 209P.

Inhibitor	Concentration, mM	Inhibition, %
1,10-Phenanthroline	1.0	41.0 ± 3.6
	2.5	84.0 ± 2.4
	5.0	100.0
p-CMB	1.0	79.0 ± 1.7
	5.0	100.0
PMSF	5.0	75 ± 0.28
	10.0	100.0
EDTA *	10.0	0
	50.0	100.0

EDTA *, Ethylenediaminetetraacetic acid.

**Table 5:** Physico-chemical properties of Blps.

Parameter	*Lysobacter* sp. IB-9374 * [25]	*L. enzymogenes* M497-1 ** [24,30]	*L. capsici* VKM B-2533^T^ *** (This Work)
pH optimum	8.0	6.5 (substrate: FAGLA) 10.0 (substrates: *S. aureus*, *M. luteus*)	9.0
Molarity optimum	ND	35 mM (substrate: *S. aureus*) 20 mM (substrate: *M. luteus*)	5 mM
Temperature optimum	65 °C	ND	50 °C
Thermal stability of enzyme/enzyme half-inactivation temperature	Up to 50 °C	ND	57 °C
pH stability	6–12	ND	4–7; 10–11
Inhibitors	Chelating and reducing agents. Insensitivity to inhibitors of serine, cysteine, aspartate proteases. Substrate: neuromidin (*L. enzymogenes*) and *M. luteus* (*Lysobacter* sp. IB-9374)		Chelating agents, inhibitors of serine and cysteine proteases
Metals	Ca ^2+^, Mg^2+^, Ba^2+^, Fe^2+^, do not affect activity Mn^2+^, Ni^2+^, Co^2+^, 30% inactivation Zn^2+^, Cd^2+^, Hg^2+^, Cu^2+^, 95% inactivation	ND	Ca^2+^, Mg^2+^, up to 30% inactivation; Mn^2+^, Ni^2+^, up to 55% inactivation; Fe^2+^, 69% inactivation; Zn^2+^, Cu^2+^, >93% inactivation

* substrate: cells of *M. luteus* IF0 3333 dried by spraying. ** substrates: cells of *M. luteus* and *S. aureus* IFO 13,276 dried by spraying. *** substrate: autoclaved cells of *S. aureus* 209P. ND, not determined.

**Table 6:** Specificities of bacteriolytic proteases compared.

Protease (Producer)	Spectrum of Bacteriolytic Action	Hydrolysis of the Bond in Peptidoglycan	Hydrolysis of the Bond in Proteins and Peptides	Ref.
Blp of *L. enzymogenes* M497-1 and ATCC29487; *Lysobacter* sp. IB-9374	Freeze-dried cells of *M. luteus* IF0 3333; *S. aureus* IAM 12544; *S. caseolyticus* ATCC 13548; *Microbacterium arborescens* JCM 5884; *B. subtilis* JCM 1465; *Arthrobacter globiformis* JCM 1332; *Enterococcus faecalis* JCM 5803; *Corynebacterium aquaticum* JCM 136; *Lactobacillus sake* JCMl157; *L. plantarum* JCM 1149; *L. casei* JCM 1134; *Lactococcus lactis* JCM 5805; *Pediococcus acidilactici* JCM 2032; *E. coli* JCM 1649; *Xanthomonas* sp. IF0 3085; *Beijiernckia indica* IF0 3744; *Enterobacter aerogenes* JCM1235	Gly-Gly, D-Ala-Gly *S. aureus*; D-Ala-L-Ala of *M. luteus* peptide bridge	Gly-Gly; Gly-Asn; Gly-Asp; Gly-His; Gly-Trp; Gly-Leu; Gly-Phe; Asn-Leu; Val-Cys	[24,25,30,33]
BlpLc	Living cells of *S. aureus* 55 (MRSA; *S. aureus* 209P; *M. luteus* B1819; *M. roseus* B1236	Not determined	Gly-Gly; Gly-Asn; Gly-His; Gly-Phe; Gly-Ala *; Gly-Lys *; Gly-Ser *;Gly-Thr *; Lys-Glu *; Glu-Leu *; Ser-Lys *; Phe-Thr *; Ala-Val *; Ala-Ala *; Ala-His *; Ala-Ser *; Val-Ser *	This work
Staphylolysin LasA *Pseudomonas aeruginosa* (Paks I, FRD2, FRD2128)	Living cells of *S. aureus* (MRSA); Freeze-dried cells of *S. saprophyticus* CECT 235; *S. epidermidis* CECT 232; *S. warneri* CECT 236; *Streptomyces griseus* CECT 3112	Gly-Gly *S. aureus*	Gly-Gly; Gly-Ala; Gly-Phe	[34,35,36]

* Hydrolysis sites in substrates (proteins, peptides) were determined for the BlpLc for the first time.

## Supplementary Materials

The following are available online at https://www.mdpi.com/2079-6382/9/11/744/s1: Figure S1: Dependence of the bacteriolytic activity of the BlpLc on some factors. (a) Temperature; (b) ionic strength; (c) pH; (d) pH stability; (e) temperature stability. Values represent means ± standard deviations of three independent measurements. Figure S2: The type of bond hydrolyzed by the BlpLc in different substrates. (a) Bovine hemoglobin, A-chain; (b) bovine hemoglobin, B-chain; (c) horse myoglobin; (d) ovamucoid; (e) ovalbumin. Arrows, bonds hydrolyzed by the BlpLc.
