# Supplementary material for: β-Lytic Protease of Lysobacter capsici VKM B-2533T
Source: Antibiotics (Basel). 2020 Oct 28;9(11):744. doi: 10.3390/antibiotics9110744 (PMC7693508; doi:10.3390/antibiotics9110744)
Supplement: Supplementary file 1 [file antibiotics-09-00744-s001.pdf]

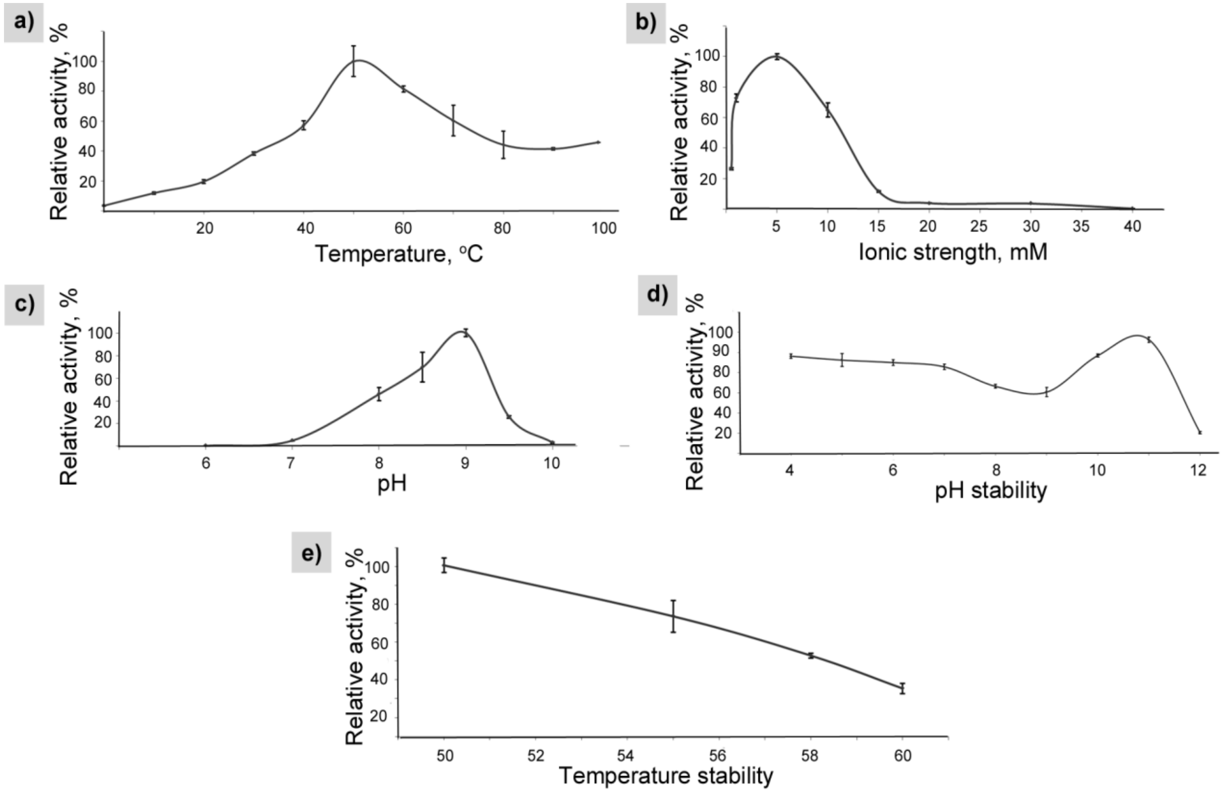

Supplementary Figure S1: Dependence of the bacteriolytic activity of the BlpLc on some factors. a) Temperature; b) ionic strength; c) pH; d) pH stability; e) temperature stability. Values represent means ± standard deviations of three independent measurements.

a)

```
          10                   20                   30                   40                   50
MVLSAADKGNVKAAWGKVGGHAAEYGAEALERMFLSFPTTKTYFPHFDLSHGSAQVK
  60                  70                   80                   90                   100                  110
GHGAKVAAALTKAVEHLDDLPGALSELSDLHAHKLRVDPVNFKLLSHSLLVTLASHLP
    120                  130                  140
SDFTPAVHASLDKFLANVSTVLTSKYR
```

b)

```
          10                   20                   30                   40                   50
MLTAEEKAAVTAFWGKVKVDEVGGEALGRLLVVYPWTQRFFESFGDLSTADAVMNNP
  60                  70                   80                   90                   100                  110
KVKAHGKKVLDSFSNGMKHLDDLKGTFAALSELHCDKLHVDPENFKLLGNVLVVVLA
       120                  130                  140
RNFGKEFTPVLQADFQKVVAGVANALAHRYH
```

c)

```
          10                   20                   30                   40                   50
MGLSDGEWQQVLNVWGKVEADIAGHGQEVLIRLFTGHPETLEKFDKFKHLKTEAEMK
  60                  70                   80                   90                   100                  110
ASEDLKKHGTVVLTALGGILKKKGHHEAELKPLAQSHATKHKIPIKYLEFISDAIIHVLH
  120                  130                  140                  150
SKHPGDFGADAQGAMTKALELFRNDIAAKYKELGFQG
```

d)

```
          10                   20                   30                   40                   50
MAMAGVFVLFSFVLCGFLPDAAFGAEVDCSRFPNATDKEGKDVLVCNKDLRPICGTDG
  60                  70                   80                   90                   100                  110
VTYTNDCLLCAYSIEFGTNISKEHDGECKETVPMNCSSYANTTSEDGKVMVLCNRAFNP
  120                  130                  140                  150                  160                  170
VCGTDGVTYDNECLLCAHKVEQGASVDKRHDGGRKELAAVSVDCSEYPKPDCTAEDR
    180                  190                  200                  210
PLCGSDNKTYGNKCNFCNAVVESNGTLTLSHFGKC
```

e)

10 20 30 40 50

MGSIGAASMEFCFDVFKELKVHHANENIFYCPIAIMSALAMVYLGAKDSTRTQINKVV

60 70 80 90 100 110

RFDKLPGFGDSIEAQCGTSVNVHSSLRDILNQITKPNDVYSFSLASRLYAEERYPILPEY

120 130 140 150 160 170

LQCVKELYRGGLEPINFQTAADQARELINSWVESQTNGIIRNVLQPSSVDSQTAMVLVN

180 190 200 210 220 230

AIVFKGLWEKAFKDEDTQAMPFRVTEQESKPVQMMYQIGLFRVASMASEKMKILELPF

240 250 260 270 280 290

ASGTMSMLVLLPDEVSGLEQLESIINFEKLTEWTSSNVMEERKIKVYLPRMKMEEKYNL

300 310 320 330 340 350

TSVLMAMGITDVFSSSANLSGISSAESLKISQAVHAAHAEINEAGREVVGSAEAGVDAAS

360 370 380

VSEEFRADHPFLFCIKHIATNAVLFFGRCVSP

Supplementary Figure S2: Type of bond hydrolyzed by BlpLc in different substrates. a) Bovine hemoglobin, A-chain; b) bovine hemoglobin, B-chain; c) horse myoglobin; d) ovamucoid; e) ovalbumin. Arrows, bonds hydrolyzed by the BlpLc.
